# Analytical Evaluation of the Dendritic Structure Parameters and Crystallization Rate of Laser-Deposited Cu-Fe Functionally Graded Materials

**DOI:** 10.3390/ma13245665

**Published:** 2020-12-11

**Authors:** Konstantin Makarenko, Oleg Dubinin, Igor Shishkovsky

**Affiliations:** Center for Design, Manufacturing and Materials, Skolkovo Institute of Science and Technology, Bolshoy Boulevard 30, build. 1, 121205 Moscow, Russia; o.dubinin@skoltech.ru (O.D.); I.Shishkovsky@skoltech.ru (I.S.)

**Keywords:** direct energy deposition, functionally graded materials, aluminum bronze, AISI 316L, Cu-Fe, dendritic structure, ellipsoid front, crystallization rate, secondary elements of dendrites

## Abstract

The paper is devoted to the direct energy deposition (DED) of functionally graded materials (FGMs) created from stainless steel and aluminum bronze with 10% content of Al and 1% of Fe. The results of the microstructure analysis using scanning electronic microscopy (SEM) demonstrate the existence of a dendritic structure in the specimens. The crystallization rate of the gradient binary Cu-Fe system structures was investigated and calculated using the model of a fast-moving concentrated source with an ellipsoid crystallization front. The width of the secondary elements of the dendrites in the crystallized slab was numerically estimated as 0.2 nm at the center point of the circle heat spot, and the two types of dendrites were predicted in the specimen: the dendrites from 0.2 to approximately 50 nm and from approximately 0.1 to 0.3 μm in width of the secondary elements. The results were found to be in good accordance with the measured experimental values of the dendritic structure geometry parameters.

## 1. Introduction

Functionally graded materials (FGMs) with a periodical or irregular gradient of phase, structure, chemical composition, physical and mechanical properties are widely implemented in the aerospace, nuclear, milling, automotive, defense, tooling, medicine, electronics/optoelectronics and many other areas of industry [1,2,3,4,5]. The choice of the concrete combination and chemical composition of the conjoining base materials in FGMs depends on the required final properties of the manufactured part or the whole assembly. It can be the composition of the materials with high mechanical strength and high heat conductivity, a base material without unique properties and materials with high antifriction characteristics (such as friction-proof bronze for bearings), high surface hardness, corrosion resistance, thermal stability and so on. Otherwise speaking, the final technical purpose and the necessary physical characteristics of the parts, assemblies or their components determine materials, phases (such as, for example, cementite, ferrite or austenite in the case of a Fe-C system) and structures (such as martensite, perlite or ledeburite of Fe-C), which form the new functionally graded material (FGM) with a gradient in an intended direction.

The first time the most important, both metallic and non-metallic, FGMs (named FGCMs—functionally graded composite materials [6]) were divided into the 16 groups was in 1995 [6,7] (Table 1, column 1). Twenty-five years after that, the authors of [4] presented the classification of FGMs in dependence on three broad groups of fabrication methods: deposition-based methods (vapor deposition, electrodeposition, thermal spray method), liquid state methods (centrifugal force methods, slip casting, tape casting, infiltration method and Langmuir–Blodgett method) and solid-state methods (powder metallurgy, friction stir welding and additive manufacturing—AM). The list of metal-based FGMs produced by the group of solid-state methods is given in Table 1, columns 2–4 [4].

The AM methods allow the cost-efficient production of parts of the FGMs with a complicated geometry in a single technological step without assembly operations, along with low waste of the material. Among these technologies for metal 3D printing are: selective laser melting (SLM), selective laser sintering (SLS), electron beam melting (EBM), wire and arc additive manufacturing (WAAM), direct energy deposition (DED) [4], also known as laser metal deposition (LMD), laser engineered net shaping (LENS^TM^) [8,9], 3D direct laser fabrication [10] or 3D laser cladding. The advantages of the last technology are higher performance in comparison with SLM, SLS, EBM and WAAM, low substrate deformation and the high automation of the process. The list of the FGMs systems fabricated through the AM methods (Table 1, column 4) [4] lacks the several important types of metallic FGMs such as Cu-Fe system FGM [11,12,13], Cu-Ni [14,15], maraging-tool steel [16] and so on. According to the results of a deep literature review, another actual classification of the majority of metal-based FGMs (in dependence on a base material) that could be produced via AM methods can be suggested as the six consolidated groups described below. A similar classification has already been represented and discussed by the authors of the current study at the international conference “RusMetalCon-2020: International Russian Conference on Materials Science and Metallurgical Technology”.

### 1.1. Ni-Based FGMs

The Ni-based FGMs are commonly built upon the nickel superalloys (such as Diamalloy 1005 [17]) which are combinations of Ni, Cr and Al. These superalloys are commonly used for tools working in elevated temperatures like airfoils of turbines [18]. Such intermetallic phases as NiAl and Ni_3_Al, which have good oxidation resistance in intense heat conditions, even under the influence of hot flows of various gases, find their application as protective coverings in aerospace and power mechanical engineering, including components of gas turbine units and engines for missiles of diverse purposes. The advantages of these materials are their moderate density, unique mechanical characteristics under high temperatures and chemical and erosion resistance within a wide temperature range [17]. Specially, the functionally graded system of Ni-Cr-B-Si and stainless steel such as SS 316L should be mentioned [19]. Ni-Cr-B-Si alloys are widely used in corrosive and high-temperature conditions. These FGMs have many different solid inclusions and are applicable for hardfacing [20].

### 1.2. Ti-Based FGMs

The Ti-based FGMs are in a similar way based on the titanium superalloys (Co-V-Ta-Ti, Co-Ni-Al-Ti, Nb-Ti, Ti-B-Cu, Ti-Al-V-C, Ti-W-C and so on), Ti and TiO_2_ [21] and aluminides of titanium. They are also widely used in aerospace and power industries, above all in high-temperature conditions [22,23]. The area of application of these FGMs is larger in comparison with pure Ti alloys. The advantages of Ti-based superalloys are higher rigidity, hardness, thermal stability and heat resistance [24,25]. The results of investigation tests performed with TiB_2_-Cu FGMs in conditions similar to a missile operation with a thermal shock influence demonstrated the absence of brittle failure or cracking [26]. Ti alloy-based functionally graded (FG) structures, as well as technically pure titanium, due to their biocompatibility, also find their application in endoprosthetics [27].

### 1.3. Al-Based FGMs

The third group is the aluminum-based FGMs. One of their common examples is a combination of AlSi40 and Al [28], where particles of Si serve as hard reinforcements of an inhomogeneous Al-based structure. The resulting material has enhanced mechanical and chemical resistance properties in comparison with initial components [28].

### 1.4. Fe-Based FGMs

The fourth important group includes, first of all, Fe-Al (such as Fe_3_Al and SS 316L), Fe-Ti and Fe-C gradient alloys. The Fe-Al compounds have many special characteristics [29]: corrosion, creep and heat resistance and significant mechanical strength in high-temperature conditions [30,31,32,33]. Manufacturing of these FGMs is not so expensive partly owing to the reasonable cost of substrates [34,35,36]. The materials of the Fe-Ti system [37,38] combine the low density, high mechanical strength and heat resistance of Ti with specific properties of steel (such as processability and lower price) [37]. The Fe-C system FGMs are represented by a combination of different kinds of steel, for example, maraging and tool steel [16] or graded SS 316L [39].

### 1.5. Cr-Based FGMs

One of the most important Cr-based FGMs is, for example, a CoCrMo multimaterial alloy. It plays an important role in biomedical applications and is used in producing implants for total knee and hip replacements (TKR and THR, respectively) [40].

### 1.6. Cu-Based FGMs 

The Cu-based FGMs are presented by, first of all, Cu-Ni (including Cu-Inconel), Cu-Al and Cu-Fe (including bronze-steel) FG systems [11,14,15]. Combining two aerospace alloys such as, for example, GRCop-84 and Inconel 718, allows enhancing the thermophysical properties of a resulting bimetallic structure [14]. Copper allows combining good oxidation resistance in aggressive atmospheres (such as alkaline and salt) with high values of heat and temperature conductivity coefficients [15]. Nickel is a common component of high-temperature alloys. Perfect notch toughness, mechanical strength and corrosion resistance in elevated temperature conditions characterize them. The high-temperature strength of Ni and heat conductivity of Cu provide usage of these FGMs in extreme temperature conditions [11]. FGMs sintered using tool steel H13 and copper can be used for purposes of the casting industry such as materials for injection molding tools due to their high mechanical strength, wear resistance and thermal conductivity [11]. The stainless steel and Cu combination finds its application in the production of food processing, steam turbine and power nuclear plants, electronic components and so on because of mutually supportive characteristics, such as the electrical and heat conductivity of copper, along with the good corrosion resistance and manufacturability of stainless steel [12].

The FGMs of group 1.6 are widely used in the manufacturing of space industry parts due to the mentioned physical and exploitation properties of copper and advantages of nickel alloys and stainless steel. The first steps in research of FGMs usage for the space industry were performed 18 years ago [26]. There is a description of FGMs application for producing thermal barrier materials for space shuttles and creating air and gas vanes, thrust chambers, piston tops, nosetips and so on. A radial laser deposition additive technology [41] allows producing axially symmetric gradients, such as metallic parts for spaceship elements created from carbonaceous filaments. Such gradient parts can be fabricated radially from the center of a sample to the outside.

Our research is devoted to the DED of the FGMs associated with group 1.6, their microstructure and crystallization characteristics: geometrical parameters of the dendrites (width of the secondary elements) and the crystallization rate of the alloy. The analytical evaluation of these parameters was performed by applying the fast-moving concentrated source model with an ellipsoid crystallization front, constant admixture distribution and insignificant diffusion of dissolved matter in a solid phase to the binary Cu-Fe system laser-deposited FGM. This mathematical model is commonly used for laser welding or laser cladding of non-gradient metals, but there is a possibility to expand its range of applicability. The authors of the current study performed the comparison of the analytical results with the experimental data and made a conclusion about the accuracy and usability of the above-specified mathematical model in practice for DED of the binary system metallic FGM. The practical significance of the conducted research in respect to Cu-Fe FGMs is discussed in Section 2: “Materials and Methods”.

Before the second section of the paper, we should briefly describe several basic terms and regularities relevant to the topic of dendritic structure forming. A dendritic structure is a crystallographic complex which has a proper orientation of axes of the single elements (the first time this definition was presented was more than 100 years ago [42]). A single dendrite consists of a trunk (primary element) and a secondary element. The secondary element of a dendrite is a branch, which grows from the trunk angularly to it; the angle between the trunk and the secondary element is commonly ≥30° and ≤90°. The admixtures and gradient of the temperature play a key role in dendritic structure forming in traditional and multimaterial structures. The degree of evolving of the higher-level axes of dendrites depends on the embedding of the crystallite (a gradient of the temperature). The development of the dendritic structure increases with the growth of the admixtures, which reduces the melting point of a solvent; the main axes of dendrites are mostly orienteered towards a heat flux direction [42]. The most common factors of dendritic structure development are associated with thermal and solutal undercooling [43].

## 2. Materials and Methods

### 2.1. Materials, Their Characteristics and Process Parameters

**Figure 1:**
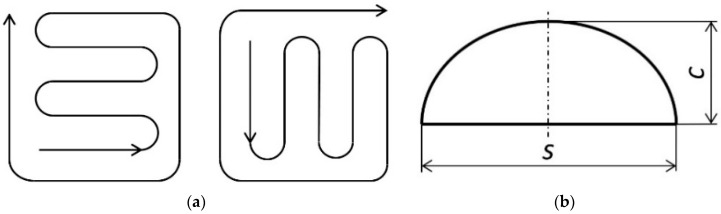
The configuration of the tracks of a single layer (even and odd layers, respectively) (**a**) and the bed profile approximate geometry (**b**).

The stainless steel AISI 316L and an aluminum bronze with 10% aluminum content doped with 1% of Fe were applied in the deposition process. Laser treatment was performed via the InssTek MX-1000 technological installation in direct metal tooling (DMT) mode. In this mode, the laser power is changed automatically from minimal to maximal value during the process according to the vision height between the sensor and the melting pool. The IPG Photonics 1 kW ytterbium fiber laser was used as a source of laser radiation. The heat spot was placed at the 1 mm level under the surface of the workpiece. Treatment was provided without preheating of the surface. The FGM was created by applying laser synthesis using the alternating layers of the binary system materials technique. This technique proved itself in our previous published research [44] and demonstrated the absence of defects such as cracking and porosity in the as-built specimens. The most important processing parameters are shown in Table 2. The patterns of the tracks within a single layer for even and odd layers and the geometrical parameters of a single bed are presented in Figure 1 and Table 2. All mentioned parameters were optimized earlier in our study [44]. The research of the microstructure was performed via the optical microscope Carl Zeiss and the scanning electronic microscope Quattro SEM. The energy-dispersive X-ray spectroscopy (EDX) analysis was performed using the Bruker maXis impact mass spectrometer (Bruker Corporation, Billerica, MA, USA).

**Figure 2:**
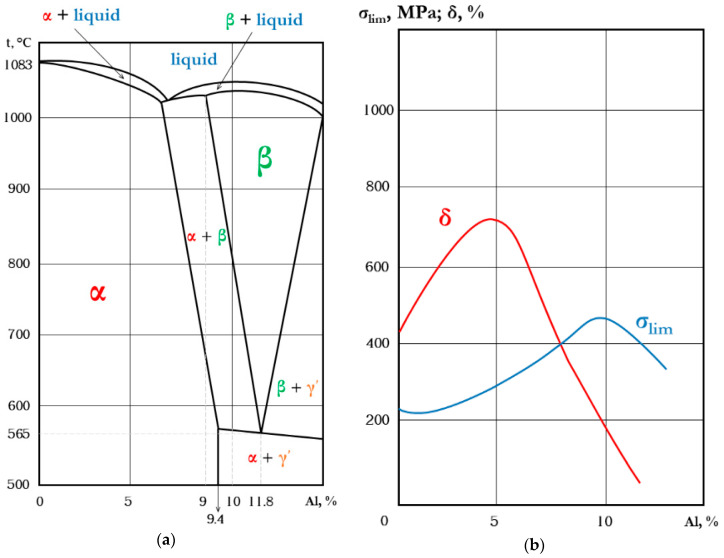
The phase diagram of aluminum bronzes (**a**) and the influence of Al on the mechanical properties of an aluminum bronze (**b**) [45].

Aluminum bronzes with more than 9% content of aluminum have the eutectoid α + γ’ in their structure at temperatures less than 565 °C (the phase diagram of the aluminum bronze is demonstrated in Figure 2a). Here, α is a solid solution of aluminum in copper and γ’ is an electron compound, Cu_32_Al_9_ [45]. The α-phase has high plasticity, but low mechanical strength; the two-phase alloys α + γ’ have higher mechanical strength, but lower plasticity (Figure 2b) [45]. The Fe doping of the aluminum bronze allows atomizing the grains and improving the mechanical and antifriction properties of the aluminum bronze. Varying the Fe concentration in aluminum bronze allows making modifications in the mentioned parameters of a resulting part. However, an unresolved issue now is the prediction of the influence of Fe in the aluminum bronze–stainless steel system laser-deposited alloy on the resulting size of grains, morphology, parameters of the dendritic structure and, finally, the mechanical properties of the gradient material.

In this light, the very promising fundamental and applied task is the possibility to predict the morphology of the laser-deposited gradient structures of the Cu-Fe system and control the resulting mechanical properties of the alloy. The problem of prediction of the dendritic structure and morphology parameters and their influence on the mechanical properties of different materials is described in many articles [46,47,48,49,50,51]. In the study of [48], the authors performed a tensile testing of Zn-Al alloys and determined the correlation between ultimate and yield strength, parameters of the solidification process and the dendrite secondary spacings. The kindred work of [51] describes dependencies between the growth of the columnar dendrites, forming of the dendritic α-Al phase and its linear relationship with mechanical properties of near-eutectic Al-Si alloys. The research of [46] is devoted to the parameters affecting the dendritic structure of as-cast aluminum alloys and the correlation between the dendritic arm spacing decrease, microstructure refinement and increase in hardness, percentage elongation, tensile strength and impact energy of the resulting structures.

### 2.2. Relevant Theory. Factors of Dendritic Structure Forming

**Figure 3:**
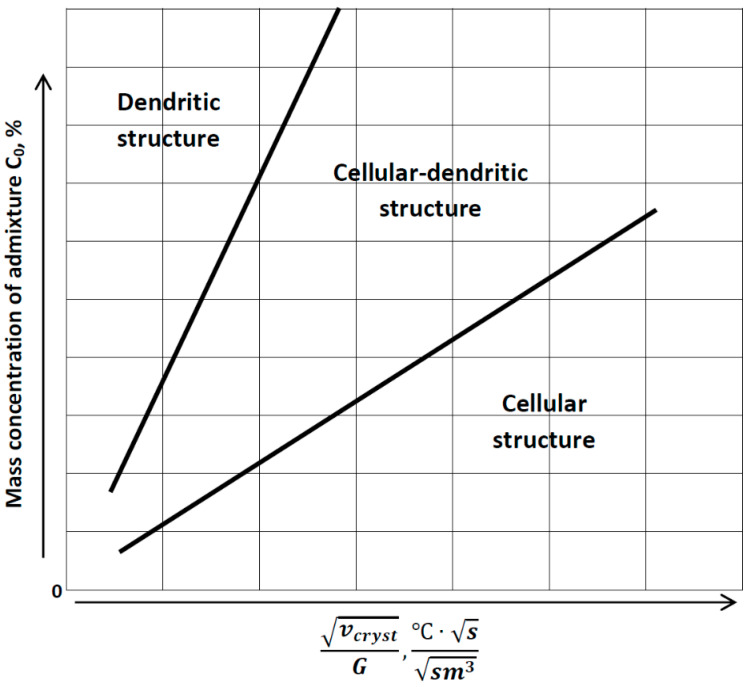
The alloy structure type dependence on the solutal undercooling criteria and the admixture concentration [52,53].

The already published studies [52,53] state that there is a dependence in different binary metal systems between the structure type (dendritic, cellular–dendritic or cellular), the solutal undercooling criteria and the admixture concentration (Figure 3) [52,53].

In Figure 3, *v_cryst_* is the crystallization rate and *G* = *∂T*/*∂n* is the temperature gradient [52] (*n* is the distance from the phase interface to the considered point towards normal direction). Figure 3 demonstrates the dependencies between the growth of the admixture amount and forming of the dendritic structure within the definite range of the solutal undercooling criteria. This dependence can be experimentally tested for binary system laser-deposited alloys such as Cu-Fe FGMs in three steps. The first step is an analytical estimation of the crystallization rate function, the second step is a determination of the temperature gradient in the volume of the workpiece and the third one is devoted to the admixture mass concentration evaluation. The current paper reports the results acquired in the first step, and our further investigation in this field is going to answer the other two questions.

### 2.3. Relevant Theory. The Diffusion of Elements through the Moving Phase Interface during the Crystallization Process and the Width of the Secondary Elements of Dendrites

During the process of metal deposition, a crystallization front moves towards the direction of a heat flux, and the crystallites grow in the direction of the maximal temperature gradient (normally to the crystallization front surface) [52]. The solubility of the admixtures in a liquid and solid metal is different; therefore, the movement of the crystallization front induces diffusion processes. Distribution of the admixtures between liquid and solid phases influences the drift of the alloy melting point. Therefore, the diffusion phenomena influence not only the chemical inhomogeneity of the deposited part, but its crystallization kinetics too.

The diffusion coefficients decrease with the transition of the metal from a liquid to solid state [52]. Therefore, the phase interface movement induces different diffusion equalizations of the admixtures in liquid and solid phases. In our further calculations, the diffusion equalization of the admixtures in the solid state is ignored because of the high crystallization rate of the laser deposition process.

Below, crystallization is considered as a linear process (movement of the flat crystallization front in a normal direction). This task was solved in [54,55] on the following assumptions:

1. Diffusion of dissolved matter in solid phase is insignificant;
2. Convection and non-diffuse mixing in liquid is important or insignificant;
3. The admixture distribution constant *k* is invariable.

We do not take into consideration the influence of the inequal temperature distribution on the diffusion fluxes of admixtures [54,55] and the influence of the phase interface distortions on admixture distribution towards the normal of this surface. In case of a laser treatment, a significant evolution of the admixtures flow normally to the isotherms is expected because of the change in the admixtures’ solubility with the changing temperature. A solution to the task of the admixtures’ intermixing in the consolidated part of the slab was solved in [54] in respect to the full intermixing of the admixtures in a liquid phase.

An increase in admixture concentration per second near the phase interface due to the movement of the crystallization front can be represented in the form

(1) $$\frac{dC_{l}}{dt}=\frac{dC_{l}}{dx}\cdot \frac{dx}{dt}=v_{cryst}\cdot \frac{dC_{l}}{dx}$$

where $C_{l}$ is the admixture distribution in a liquid phase near the crystallization front, and $v_{cryst}$ is the crystallization rate [55].

The process was considered to be steady-state; therefore, the amount of the admixture that elbows out is equal to the amount of the admixture, which diffuses in liquid. The concentration distribution near the moving phase interface is constant in time: *dC_l_*/*dt* = 0. This assumption can be written as a differential equation:

(2) $$\frac{dC_{l}}{dt}=D\cdot \frac{d^{2}C_{l}}{d{\left(x^{\prime }\right)}^{2}}+v_{cryst}\cdot \frac{dC_{l}}{dx^{\prime }}=0$$

where *x*′ is a coordinate in a moving coordinate system associated with the shifting phase interface, and *D* is the diffusion coefficient. The resulting admixture distribution (see Appendix A) is as follows:

(3) $$C_{l}=C_{0}\cdot \left(1+\frac{1-k}{k}\cdot e^{-\frac{v_{cryst}}{D}\cdot x^{\prime }}\right)$$

It is seen from (3) that $C_{l}=\frac{C_{0}}{k}$ if *x*′ = 0 and the concentration exponentially falls down up to $C_{l}=C_{0}$ if $x^{\prime }=\infty .$

The criterion of the exponent increases with the growth of the crystallization rate, so the admixture concentration decreases, more rapidly moving away from the phase interface.

The value of the gradient *dC*/*dx*′ determines a fall in the admixture concentration. Derivation of Equation (3) shows

(4) $$\frac{dC_{l}}{dx\prime }=C_{0}\cdot \frac{k-1}{k}\cdot \frac{v_{cryst}}{D}\cdot e^{-\frac{v_{cryst}}{D}\cdot x^{\prime }}$$

It is seen from (4) that the concentration gradient has its ultimate value at the point $x^{\prime }=0$ and then falls down up to 0 if $x^{\prime }=\infty .$ The intensity of the admixture concentration decrease increases with the growth of the crystallization rate.

The thickness of the admixture concentrating densification layer near the phase interface according to (4) is

(5) $$\delta =x^{\prime }=\frac{D}{v_{cryst}}\cdot ln\frac{C_{0}\cdot \left(1-k\right)}{k\cdot \left(C_{l}-C_{0}\right)}$$

For a first approximation, according to (5), the half-width of the secondary element (secondary branch) of the dendrite may be assumed equal to the width of the concentration seal *n_y_*:

(6) $$n_{y}=\frac{D_{l}}{v_{cryst}}=\frac{a}{2}$$

so

(7) $$a=\frac{2\cdot D_{l}}{v_{cryst}}$$

where *D_l_* is a diffusion coefficient in a liquid phase of the alloy. After estimation of the crystallization rate and the diffusion coefficient, the approximate value of *a* can be found and compared with the results of the experiments.

### 2.4. Relevant Theory. The Crystallization Rate

**Figure 4:**
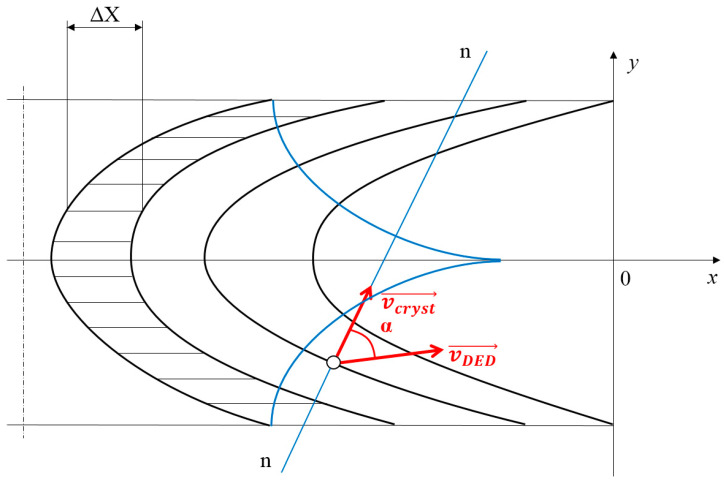
The illustration of the mutual bracing of the vectors $\vec{v_{cryst}}$ and $\vec{v_{DED}}$ [52].

In every point of the crystallization front, the vector of the crystallization rate is normal to it [52]. The vector $\vec{v_{cryst}}$ can be found from the equation

(8) $$\vec{v_{cryst}}=\vec{v_{DED}}\cdot \cos\alpha$$

where *α* is an angle between a vector of a scanning speed $\vec{v_{DED}}$ and a vector of the crystallization rate $\vec{v_{cryst}}$ (as it is mentioned above, it is normal to the crystallization front (Figure 4)) [52].

**Figure 5:**
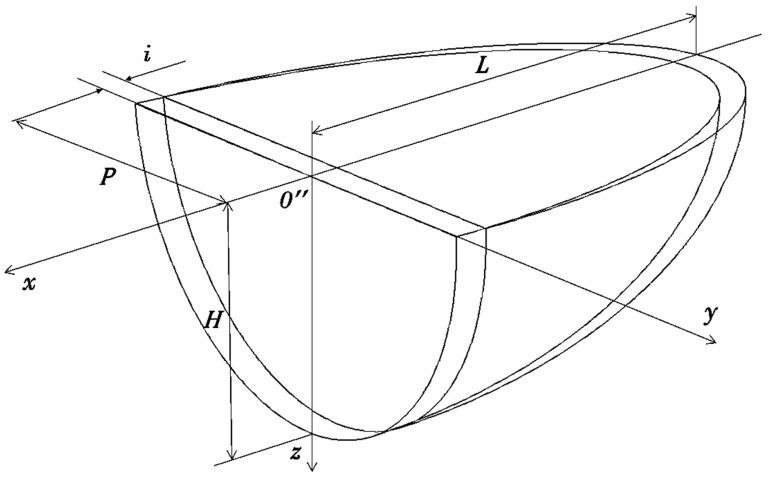
The moving ellipsoid crystallization front scheme.

The most common types of the crystallization front surfaces classes can be divided into the three groups: paraboloid surfaces classes, conic surfaces classes and classes of surfaces with a knuckle line [52]. Our model was assumed as the most widespread paraboloid-type surface class with the equation

(9) $$F\left(x,y,z,i\right)=\frac{x+i}{L}+\frac{y^{2}}{P^{2}}+\frac{z^{2}}{H^{2}}-1=0$$

where *i* is a shift of the surface class towards the *O″x* axis (Figure 5). Our assumption about the paraboloid surface type is spontaneous to a significant degree but allows us to make a good estimation for a first approximation. As it will be demonstrated below, our choice provided accurate results, proved by comparison with the experimental data.

A class of crystallization fronts can be submitted from (9) in a form of ellipsoids:

(10) $$x=f\left(y,z\right)=L\cdot \sqrt{1-{\left(\frac{y}{P}\right)}^{2}-{\left(\frac{z}{H}\right)}^{2}}-i$$

The angle *α* between the *O″x* and a normal to the crystallization front surface in any point can be defined by

(11) $$\cos\alpha =\frac{1}{\sqrt{1+{\left(\frac{\partial f\left(y,z\right)}{\partial y}\right)}^{2}+{\left(\frac{df\left(y,z\right)}{dz}\right)}^{2}}}$$

From Equation (10)

(12) $$\frac{\partial f\left(y,z\right)}{\partial y}=-\frac{L}{P^{2}}\cdot \frac{y}{\sqrt{1-{\left(\frac{y}{P}\right)}^{2}-{\left(\frac{z}{H}\right)}^{2}}}$$

(13) $$\frac{\partial f\left(y,z\right)}{\partial y}=-\frac{L}{H^{2}}\cdot \frac{z}{\sqrt{1-{\left(\frac{y}{P}\right)}^{2}-{\left(\frac{z}{H}\right)}^{2}}}$$

## 3. Results

**Figure 6:**
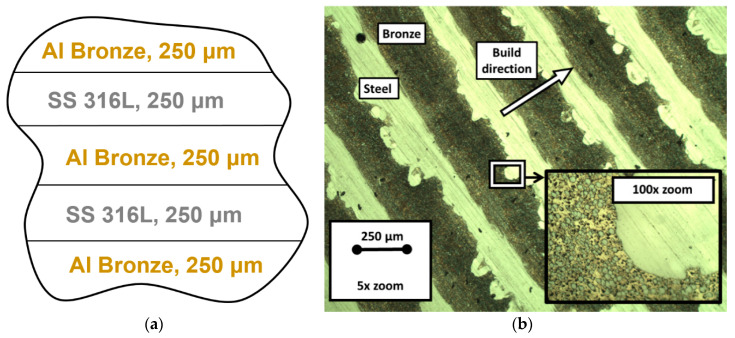
The laser deposition scheme (**a**) and the resulting gradient structure (**b**).

**Figure 7:**
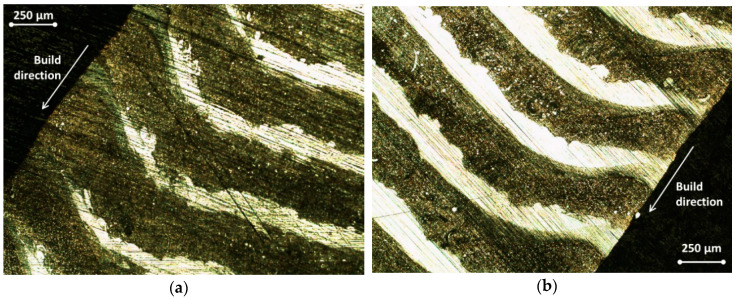
Edge effects in the gradient specimens (**a**,**b**). As in Figure 6, the dark layers correspond to bronze, and the light layers correspond to steel.

The scheme of the multimaterial deposition is shown in Figure 6a; the resulting microstructure with alternating layers of SS 316L and aluminum bronze optical micrograph is demonstrated in Figure 6b. All images of the microstructure demonstrate the absence of cracking or visible porosity in the whole volume of the specimen. We also observed the interpenetration of steel and bronze between nearby layers in the case of steel-on-bronze deposition, but the bronze-on-steel gives us a sharp and clear interface, as it is seen in Figure 6b. The aluminum bronze forms a cross-linked morphology, which is seen in the ×100 magnified image. The angle deformations associated with inequal overheating were observed in the edge areas of the specimen (Figure 7).

**Figure 8:**
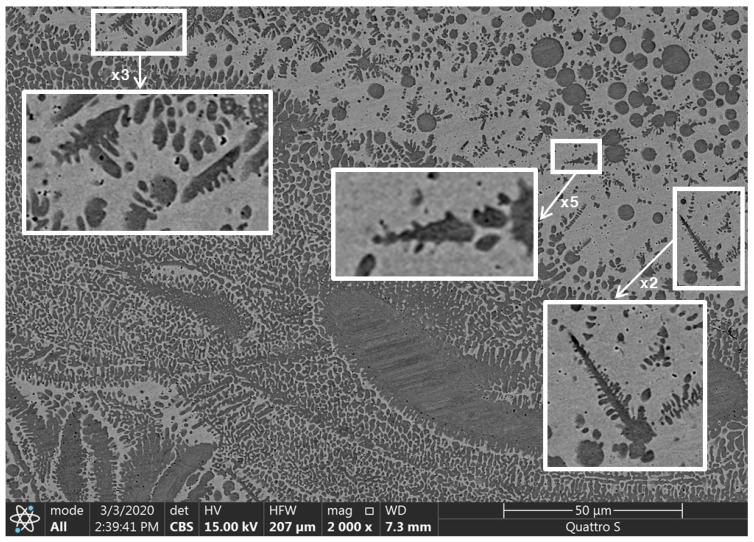
The dendritic structure of the functionally graded specimen.

**Figure 9:**
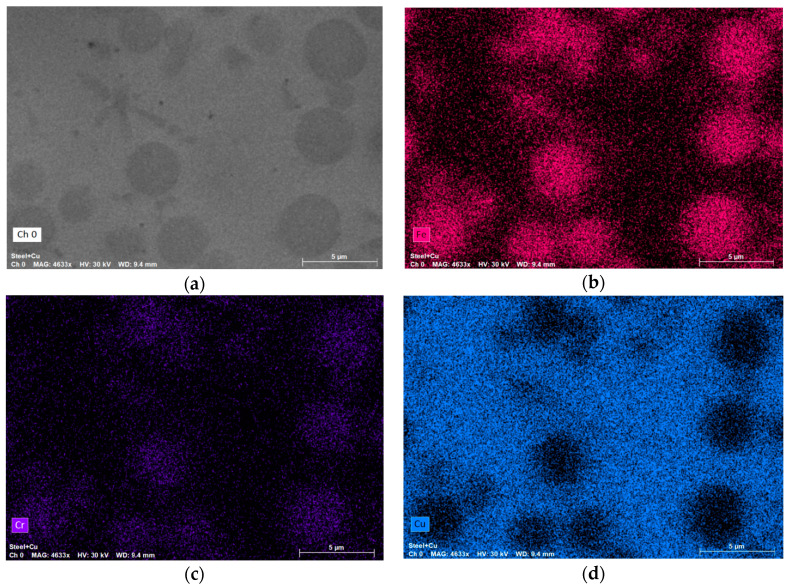
The results of EDX analysis ((**a**)—the base image, (**b**)—the Fe-based regions, (**c**)—the Cr-based regions, (**d**)—the Cu-based regions).

The results of the scanning electronic microscopy, which prove the presence of the dendritic structure, are shown in Figure 8; the inserts demonstrate the single dendrites. The dark areas in Figure 8 are related to the Fe-Cr-based regions; light areas are related to the Cu-based regions (see the results of the XRD analysis in Figure 9; the other elements, such as C, Al and Ni, were distributed almost regularly within the entire area of scanning). The significant inequality of the Fe-Cr-based structural elements size is seen in the SEM images. Different regions of the specimen demonstrated different microstructures: Fe-based elements have spherical and quasispherical shapes in one region and columnar and dendritic forms in the other. The ultra-high disproportional dendritic-shape crystal is observed in the right-bottom area of the image (Figure 8). The average width of its primary element is approximately equal to 18 μm.

**Figure 10:**
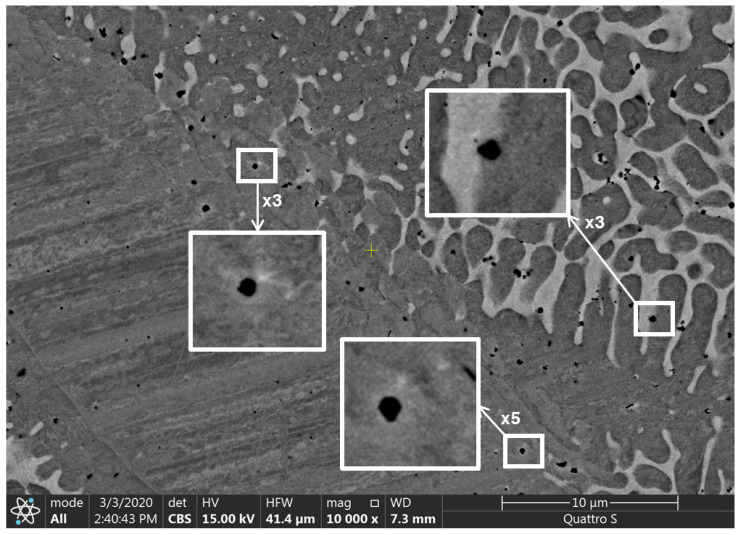
The sub-microscale porosity of the specimen.

The dendritic structure that occurred during the crystallization process of the FGM in our case was observed mainly in the central part of the slab. It was an area where the temperature gradient near the crystallization front had its minimal value, which is in good accordance with the theory [52]. It is known that the most common occurrence of dendrites is a shrinkage porosity [52]. In our case, all specimens were replete with sub-microscale pores (size of a single pore was less than 500 nm) (Figure 10), and the areas with the highest porosity consisted of the highest amount of dendrites.

**Figure 11:**
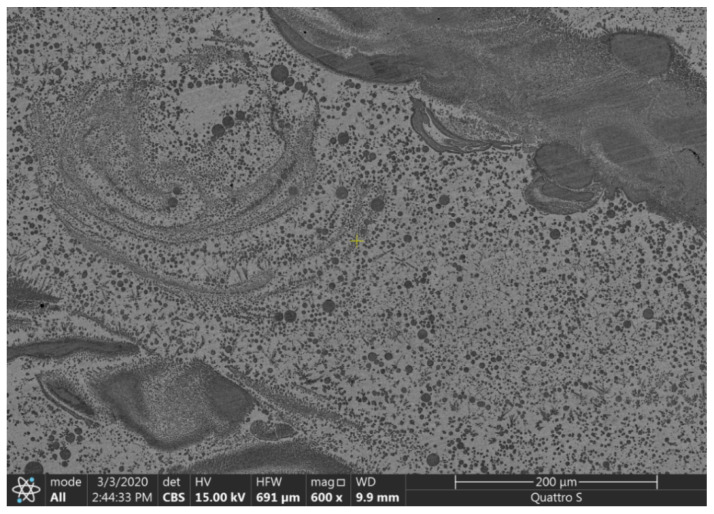
An SEM micrograph of the Cu-Fe FGM.

The dendritic and spherical cellular structure is also seen in the micrograph in Figure 11. The interesting phenomenon here is an inner curvature inside the Cu-based region (a large light area in the middle of the image) that demonstrates a solidified turbulent whirlwind-like flow of the liquid alloy.

**Figure 12:**
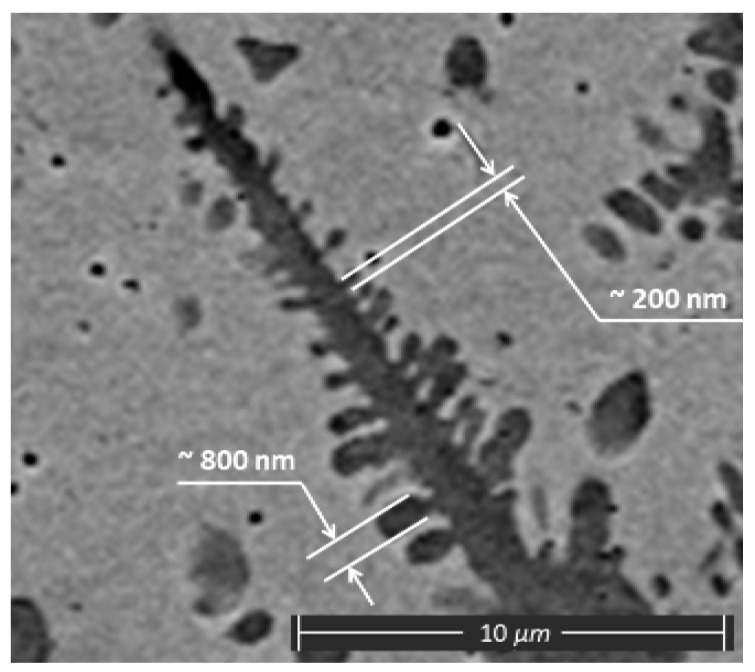
The evaluation of the secondary elements of the dendrites sketch.

The size of the single dendrite secondary elements was estimated using the results of SEM (Figure 12).

## 4. Discussion

Let us write Equations (12) and (13) (Section 2.4) in the dimensionless coordinates $K_{y}=y/P,$ $K_{z}=z/H$:

(14) $$\frac{\partial f\left(y,z\right)}{\partial y}=-\frac{K_{y}}{K_{PL}\cdot \sqrt{1-K_{y}^{2}-K_{z}^{2}}}$$

(15) $$\frac{\partial f\left(y,z\right)}{\partial z}=-\frac{K_{z}}{K_{HL}\cdot \sqrt{1-K_{y}^{2}-K_{z}^{2}}}$$

where $K_{PL}=L/P$ and $K_{HL}=L/H$ are the dimensionless form factors. Here, *P* is the half-width of a single track (Figure 1), and *H* is the depth of the laser influence (the depth of the heat affected zone—HAZ). According to the “Materials and Methods”:

(16) $$P=\frac{800}{2}=400\;\mu m=4\times 10^{-4}\;m$$

The depth of the HAZ *H* can be estimated from [56,57]

(17) $$H≅2\cdot \sqrt{\frac{a\cdot \tau_{p}}{\pi }}-\frac{T_{melt}\cdot \lambda }{q}$$

where $\tau_{p}$ is the duration of the laser treatment (under the condition $\tau_{p}\leq \tau_{max}$, where $\tau_{max}=d_{f}^{w}/\left(2\cdot a\right)$ and $d_{f}^{w}$ is the laser beam diameter on the surface of the workpiece [56]), and q is the laser power density on the surface of the workpiece. In our conditions, the average laser power $Q_{AB}=500\;W$ for aluminum bronze, and $Q_{SS}=308\;W$ for stainless steel (see “Materials and Methods”). The average laser power for our calculations is assumed equal to

(18) $$Q=\sqrt{Q_{AB}\cdot Q_{ss}}≅392\;W$$

**Figure 13:**
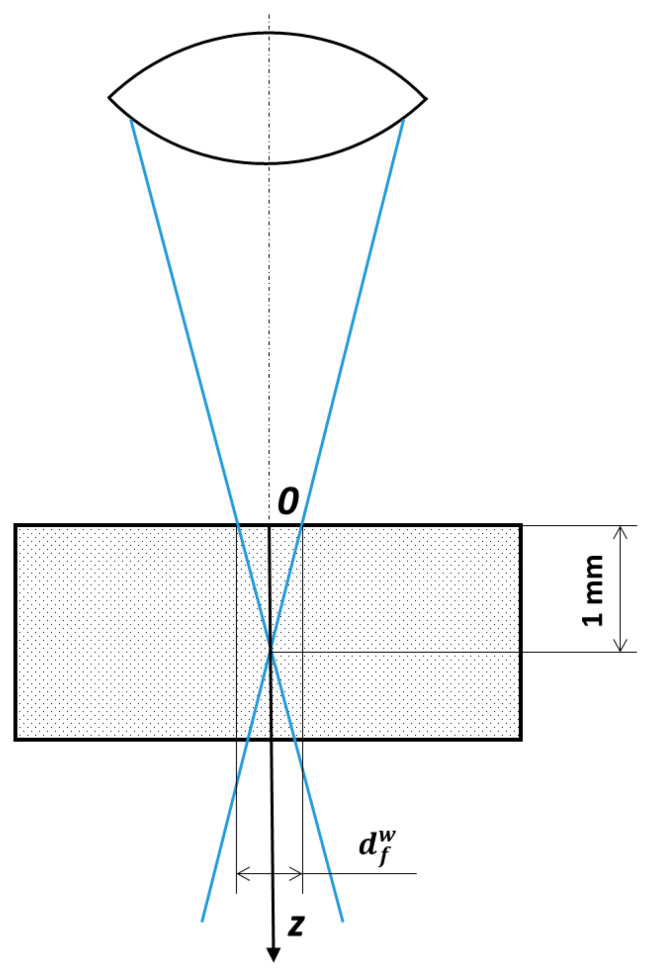
The laser source focusing scheme.

In our experiments, the approximate diameter of the spot on the surface of the workpiece was estimated as $d_{f}^{w}=500\;\mu m$ (Figure 13), so the average laser power density on the surface of the workpiece is

(19) $$q=\frac{4\cdot Q}{\pi \cdot {\left(d_{f}^{w}\right)}^{2}}≅2.00\times 10^{9}\;W/m^{2}$$

Near the melting points, with the atmosphere pressure, the thermal diffusivity of SS 316L is approximately $a_{SS}≅3.5\times 10^{-6}\;m^{2}/s$, of aluminium bronze $a_{AB}≅1.5\times 10^{-4}\;m^{2}/s$ and of air $a_{air}≅3.6\times 10^{-4}\;m^{2}/s$. Since the experiments were performed with a binary alloy of both metals with a small amount of porosity, the generalized thermal diffusivity factor was estimated for a first approximation as

(20) $$a=a_{air}\cdot \frac{V_{air}}{V_{\sum }}+a_{AB}\cdot \frac{V_{AB}}{V_{\sum }}+a_{SS}\cdot \frac{V_{SS}}{V_{\sum }}≅7.7\times 10^{-5}\;m^{2}/s$$

Here, approximately, the volume of air was estimated as 0.1%, of bronze 49.95% and of steel 49.95% of the whole slab.

The same calculation should be provided with a heat conductivity coefficient: $\lambda_{SS}≅35.1\;W/\left(m\cdot K\right),$ $\lambda_{AB}≅75.0\;W/\left(m\cdot K\right)$, $\lambda_{air}≅0.1\;W/\left(m\cdot K\right)$:

(21) $$\lambda =\lambda_{air}\cdot \frac{V_{air}}{V_{\sum }}+\lambda_{AB}\cdot \frac{V_{AB}}{V_{\sum }}+\lambda_{SS}\cdot \frac{V_{SS}}{V_{\sum }}≅55.0\;W/\left(m\cdot K\right)$$

The melting points of our metals are approximately equal to: $T_{melt}^{SS}≅1420\;{}^{\circ }C$ and $T_{melt}^{AB}≅1070\;{}^{\circ }C$; the metal with the lower melting point decreases the melting point of the whole alloy [53]; and the reason for the drift of the melting point of the binary alloy is the distribution of the admixtures between liquid and solid phases (as it was mentioned in the beginning of Section 2.3). Therefore, for a first approximation, the melting point of our binary alloy can be considered as

(22) $$T_{melt}=\frac{T_{melt}^{SS}+T_{melt}^{AB}}{2}≅1245\;{}^{\circ }C=1518.15\;K$$

In our case, the laser pulse duration was set as

(23) $$\tau_{p}=0.001\cdot \tau_{p}^{max}=\frac{d_{f}^{w}}{2000\cdot a}=1.79\times 10^{-3}\;s$$

Therefore, from (17)

(24) $$H≅8.83\times 10^{-5}\;m$$

So

(25) $$K_{y}=\frac{y}{P}=2500\cdot y$$

(26) $$K_{z}=\frac{z}{H}≅11325\cdot z$$

Assuming (11), (14) and (15),

(27) $$\cos\alpha =\frac{1}{\sqrt{1+\frac{{\left(\frac{K_{y}}{K_{PL}}\right)}^{2}+{\left(\frac{K_{z}}{K_{HL}}\right)}^{2}}{1-K_{y}^{2}-K_{z}^{2}}}}$$

If we paste

(28) $$K_{LP}^{2}=\frac{1}{K_{PL}^{2}}=\frac{L^{2}}{P^{2}}=\frac{\frac{l^{2}\cdot B^{2}}{2\cdot B-1}}{\frac{p^{2}\cdot B^{2}}{2\cdot B-1}}=\frac{l^{2}}{p^{2}}=\frac{{\left(e-1\right)}^{2}}{8\cdot \pi \cdot e}\cdot \frac{n^{2}}{m^{2}}\cdot \frac{q\cdot v_{DED}}{a\cdot \lambda \cdot T_{melt}}=0.043217\cdot \frac{n^{2}}{m^{2}}\cdot \frac{q\cdot v_{DED}}{a\cdot \lambda \cdot T_{melt}}$$

and

(29) $$\frac{1}{K_{HL}^{2}}=\frac{1}{K_{PL}^{2}}\cdot \frac{j^{2}}{n^{2}\cdot \left(2\cdot B-1\right)}$$

into (27), the dependence of the crystallization rate from the deposition conditions with a fast-moving concentrated source will be found in the case of the ellipsoid crystallization front:

(30) $$v_{cryst}=v_{DED}\cdot \cos\alpha =\frac{v_{DED}}{\sqrt{1+0.043217\cdot \frac{q\cdot v_{DED}}{a\cdot \lambda \cdot T_{melt}\cdot m^{2}}\cdot \frac{n^{2}\cdot K_{y}^{2}+\frac{j^{2}}{2\cdot B-1}\cdot K_{z}^{2}}{1-K_{y}^{2}-K_{z}^{2}}}}$$

If the crystallization front is described as a full ellipsoid, then *B* = 1, and *m* = *n* = *j* = 1 [52]. In this light,

(31) $$v_{cryst}\left(y,z\right)=v_{DED}\cdot \cos\alpha ≅\frac{v_{DED}}{\sqrt{1+0.00672\cdot q\cdot v_{DED}\cdot \frac{K_{y}^{2}+K_{z}^{2}}{1-K_{y}^{2}-K_{z}^{2}}}}$$

(32) $$v_{cryst}\left(y,z\right)≅\frac{0.014}{\sqrt{1+0.00672\times 2\times 10^{9}\times 0.014\times \frac{\left(6.25\cdot y^{2}+128\cdot z^{2}\right)\times 10^{6}}{1-\left(6.25\cdot y^{2}+128\cdot z^{2}\right)\times 10^{6}}}}\;≅\frac{0.014}{\sqrt{1+\frac{1.9\times 10^{11}\cdot \left(6.25\cdot y^{2}+128\cdot z^{2}\right)}{1-\left(6.25\cdot y^{2}+128\cdot z^{2}\right)\times 10^{6}}}}$$

Now, the value of *v_cryst_* in any point (*y*, *z*) that belongs to the ellipse segment can be estimated:

(33) $$\{\begin{array}{c} {\left(\frac{y}{P}\right)}^{2}+{\left(\frac{z}{H}\right)}^{2}\leq 1\\ 0\leq z\leq H \end{array}$$

among the research of the function behavior in the irregular points.

In the middle point (the center of a laser beam spot) *y* = 0, *z* = 0, the crystallization rate is equal to the laser scanning speed according to (32):

(34) $$v_{cryst}\left(0,0\right)≅\frac{0.014}{\sqrt{1+\frac{0}{1}}}=0.014\;m/s=v_{DED}$$

In general, this result could be achieved directly from (30): independently of concrete values of the process parameters, the crystallization rate in point (0, 0) is equal to the scanning speed of the source.

**Proposition** **1.** *Using (32) (in particular) or (30) (in general), the proposition about the crystallization rate in the crystallization front can be proved:*

(35) $$\underset{z\to H}{\lim}v_{cryst}\left(0,z\right)=\underset{y\to P}{\lim}v_{cryst}\left(y,0\right)=0.$$

**Proof** **of** **Proposition** **1.** Firstly, in our case, in the border point *y* = 0, *z* = *H* = 8.83 × 10^−5^, from (32)

(36) $$v_{cryst}\left(0,H\right)≅\frac{0.014}{\sqrt{1+\frac{1.9\times 10^{11}\times \left(0+128\times 7.8\times 10^{-9}\right)}{1-\left(0+128\times 7.8\times 10^{-9}\right)\times 10^{6}}}}≅\frac{0.014}{\sqrt{1+\frac{1.9\times 10^{5}}{1-1}}}≅\frac{\mathrm{const}}{\sqrt{1+\infty }}$$

(37) $$\underset{z\to H}{\lim}v_{cryst}\left(0,z\right)=\frac{1}{\sqrt{1+\infty }}=\frac{1}{\sqrt{\infty }}=\frac{1}{\infty }=0$$

Secondly, in the other border point *y* = *P* = 4 × 10^−4^, *z* = 0, from (32)

(38) $$v_{cryst}\left(P,0\right)≅\frac{0.014}{\sqrt{1+\frac{1.9\times 10^{11}\times \left(6.25\times 1.6\times 10^{-7}+0\right)}{1-\left(6.25\times 1.6\times 10^{-7}+0\right)\times 10^{6}}}}≅\frac{0.014}{\sqrt{1+\frac{1.9\times 10^{5}}{1-1}}}=\frac{\mathrm{const}}{\sqrt{1+\infty }}$$

(39) $$\underset{y\to P}{\lim}v_{cryst}\left(y,0\right)=\frac{1}{\sqrt{1+\infty }}=\frac{1}{\sqrt{\infty }}=\frac{1}{\infty }=0=\underset{z\to H}{\lim}v_{cryst}\left(0,z\right)$$

 □

The melting process stops by reaching the crystallization front, so the crystallization rate in these points becomes equal to zero in the limit, and this phenomenon does not depend on the concrete parameters of process, which can be easily proved using (30).

**Figure 14:**
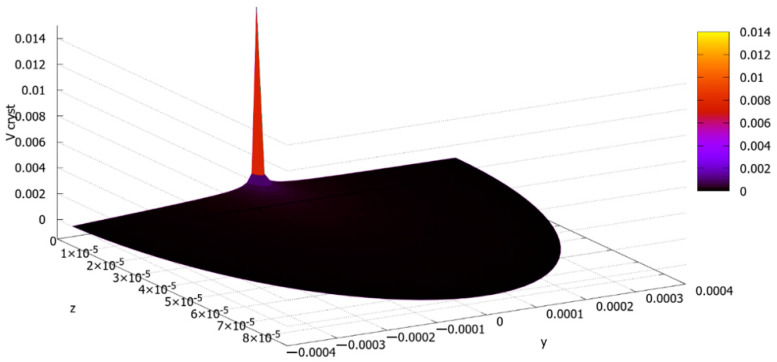
The crystallization rate function graph ([*z*] = *m*, [*y*] = *m*, [*v_cryst_*] = m/s).

Figure 14 demonstrates the graph of the two-variable function *v_cryst_* (*y*, *z*). It is seen that the value $v_{cryst}=v_{DED}=0.014\;m/s$, which was calculated above, is a maximal value of this function (and belongs to point (0, 0)), and the crystallization rate steeply decreases with the distance from the center of the laser heat spot.

### The Final Calculations and Their Discussion

Now, using the function *v_cryst_* (*y*, *z*), the width of the secondary elements of the dendrites function *a* (*y*, *z*) can be defined and the size *a* (*y*, *z*) in any point of the area (33) can be found:

(40) $$a\left(y,\;z\right)=\frac{2\cdot D_{l}}{\frac{0.014}{\sqrt{1+\frac{1.9\times 10^{11}\times \left(6.25\cdot y^{2}+128\cdot z^{2}\right)}{1-\left(6.25\cdot y^{2}+128\cdot z^{2}\right)\times 10^{6}}}}}=\frac{D_{l}\cdot \sqrt{1+\frac{1.9\times 10^{11}\times \left(6.25\cdot y^{2}+128\cdot z^{2}\right)}{1-\left(6.25\cdot y^{2}+128\cdot z^{2}\right)\times 10^{6}}}}{0.007}.$$

For the Cu-Fe system, the diffusion coefficient was estimated in [58] as

(41) $$D_{l}=0.03\times 10^{-4}\cdot e^{\frac{-187\times 10^{3}}{R\cdot T}}$$

In our case, *T* = *T*_melt_ = 1518.15 K, and *R* = *const* ≅ 8.315 J/(mol·K). Therefore,

(42) $$D_{l}=0.03\times 10^{-4}\cdot e^{\frac{-187\times 10^{3}}{8.315\times 1518.15}}≅1.12\times 10^{-12}\;m^{2}/s$$

Firstly, in can be seen that the width of the secondary elements of the dendrites at the center of the laser source spot (0, 0) depends only on the laser scanning speed $v_{DED}$ (34) and the diffusion coefficient $D_{l}$:

(43) $$a\left(0,\;0\right)=\frac{D_{l}\cdot \sqrt{1+\frac{0}{1}}}{0.007}≅1.6\times 10^{-10}\;m≅0.2\;\mathrm{nm}=\frac{2\cdot D_{l}}{v_{cryst}\left(0,\;0\right)}=\frac{2\cdot D_{l}}{v_{DED}}$$

Therefore, from (40) and (42)

(44) $$a\left(y,\;z\right)=1.6\times 10^{-10}\times \sqrt{1+\frac{1.9\times 10^{11}\times \left(6.25\cdot y^{2}+128\cdot z^{2}\right)}{1-\left(6.25\cdot y^{2}+128\cdot z^{2}\right)\times 10^{6}}}$$

The width of the secondary elements of the dendrites is in inverse proportion with the crystallization rate (7). While the crystallization rate decreases, the secondary elements become wider. In the border points (*P*, 0) and (0, *H*), where the crystallization rate reaches zero in limit (35), the width of the secondary elements of the dendrites function reaches the infinite value in limit

(45) $$\underset{y\to P}{\lim}a\left(0,z\right)=\underset{z\to H}{\lim}a\left(y,0\right)=\frac{const}{0}=\infty$$

**Figure 15:**
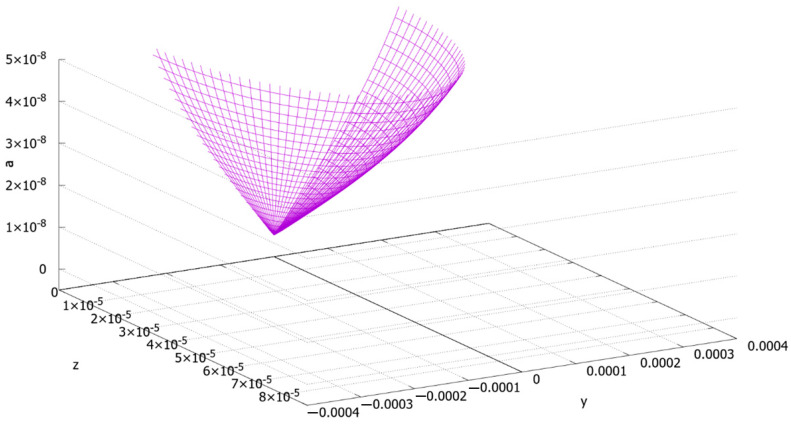
The area of the “small dendrites” ([*z*] = [*y*] = [*a*] = *m*).

**Figure 16:**
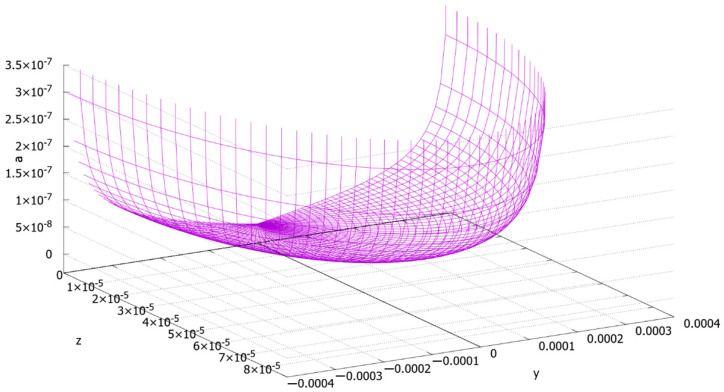
The width of the secondary elements of the dendrites function graph ([*z*] = [*y*] = [*a*] = *m*).

Function (44) demonstrates that there are two approximate theoretical areas of the dendrites in our specimen. The first area (Figure 15) is the region of the nanoscale and quasinanoscale (~50 nm) dendrites (“small dendrites”). The minimal value of the function is reached at point (0, 0) (as it was calculated above, it is equal to 0.2 nm). The second area is the region of the “huge dendrites”, where the secondary elements of dendrites reach the submicronic size (approximately from 10^−7^ to 3 × 10^−7^ m). Figure 16 demonstrates the function behavior for interval 0 ≤ *a* ≤ 3.5 × 10^−7^ m. It is seen from Figure 16 that there is a steep increase in *a* by getting closer to the crystallization front. It is evident that point *a* = *a*_max_, aligned with the hugest dendrites of the specimen, should exist. The authors will estimate this value numerically and experimentally in further research.

It can be seen from Figure 12 (Section 3) that the numerical results have good accordance with the experimental data in the area of the “huge dendrites”. The width of the secondary elements of the dendrites from Figure 12 was estimated as being from 200 to 800 nm (from 2 × 10^−7^ to 8 × 10^−7^ m).

In conclusion, it should be mentioned that there is a theoretical dependence between the width of a secondary element of a dendrite and the temperature gradient [52]:

(46) $$a=\frac{2∆T\cdot \mathrm{tg}\gamma }{G}$$

where γ is the vertex angle of the element of the dendrite, and ∆T is the crystallization interval of the alloy. Using Equation (7), the system (49) for *γ* can be achieved:

(47) $$\frac{2\cdot D_{l}}{v_{cryst}}=\frac{2∆T\cdot \mathrm{tg}\gamma }{G}$$

(48) $$\mathrm{tg}\gamma =\frac{G\cdot D_{l}}{v_{cryst}\cdot ∆T}$$

(49) $$\{\begin{array}{c} \gamma =\mathrm{arctg}\left(\frac{G\cdot D_{l}}{v_{cryst}\cdot ∆T}\right)\\ -\frac{\pi }{2}<\gamma <\frac{\pi }{2} \end{array}$$

The estimation of the vertex angles of the dendrites growing in the Cu-Fe FGMs using (49) and comparison of the analytical values with the angles measured during experiments is a viable task for future research in this field.

## 5. Conclusions

The authors performed the DED of an FGM created from aluminum bronze with 10% Al and 1% Fe content and SS 316L using the alternating layers technique [44], observed the microstructure using optical and SEM methods and performed its EDX analysis. Using the fast-moving point source model with an ellipsoid crystallization front, two important crystallization parameters were analytically evaluated: the crystallization rate and the width of the secondary elements of the dendrites. The analytical dependence between vertex angles of the dendrites, the crystallization rate, crystallization interval and temperature gradient was demonstrated. It was shown that in the case of a model of a fast-moving concentrated source with an ellipsoid crystallization front, the crystallization rate at the center point of the heat spot is equal to the laser scanning speed. The width of the secondary elements of the dendrites *a* in this point of the Cu-Fe laser-deposited FGM was evaluated using the model as 0.2 nm. The function *a* (*y*, *z*) demonstrated the existence of an area of “small dendrites” (with the width of the secondary elements from 0.2 to approximately 50 nm) and “huge dendrites” (approximately from 0.1 to 0.3 μm) (these results were calculated using the resulting equation). The theoretical width of the secondary elements of the “huge dendrites” was found to be in good accordance with the experimental data (it was estimated as being from 0.2 to 0.8 μm using the SEM micrographs). Near the crystallization front, the crystallization rate tends to zero, and the width of the secondary elements of the dendrites tends to infinity, according to the results of the theoretical analysis. The authors suppose that there are no dendrites in the FGM after the “critical point”, whose coordinates (*y*_max_, *z*_max_) will be estimated in future studies. The results of our research can be applied in further investigations of the crystal structure of laser-deposited binary system FGMs, for improvement of the mathematical models and methods in this area of the crystallization process physics. The possibility to predict the geometrical parameters of the micro- and nanostructure at the single-crystal level allows varying the necessary physical and mechanical characteristics of the resulting gradient materials [46,47,48,49,50,51].

## Figures and Tables

**Table 1:** The classifications of functionally graded composite materials (FGCMs) and solid-state methods-produced functionally graded materials (FGMs) [4,6,7].

FGCMs Systems [6,7]	FGMs Systems Fabricated through Powder Metallurgy Method [4]	FGMs Systems Developed via Friction Stir Welding (FSW) Method [4]	FGMs Systems Fabricated through the AM Methods (LMD/DED, SLM) [4]
SiC-SiC	Ni-Ti_3_AlC_2_	AA5083-Al_2_O_3_&SiC_p_	Ti6Al4V-TiC_p_
Al-SiC	Al2124-SiC	Pure Al-TiC_p_	Ti6Al4V-AlSi10Mg
SiCw-Al-alloy	Al2024-SiC	AA5083-SiC_p_	Ti-Al alloys
E-glass-Epoxy	Ni-Al_2_O_3_	AA6082 T6-SiC_p_	SS 304L-Inconel 625
Al-C	Al-Steel	Al 6061-SiC_p_	Ti6Al4V-Mo
Al-SiC	Al-B_4_C	AA6061 T6-SiC_p_	Fe-Cr-Ni alloy
SiCp-Al-alloy	SS 316-HA	Pure Al-Al_2_O_3_ & TiC_p_	Ti6Al4V-TiCp
Carbon and glass fibers	SS 316L-CS	-	SS 316L-P21
Glass-Epoxy	Al2124-Al_2_O_3_	-	Ti6Al4V-Invar
TiAl-SiC fibers	ZrO_2_-Ni	-	Ti6Al4V-SS 304L-V
Be-Al	Cu-NbC	-	Ni-Cr-B-Si-SS 316L
Al_2_O_3_-Al-alloy	ZrO_2_-NiCr	-	SS 316L-IN625
Carbon-Bismaleimide	Al-SiC	-	Ti6Al4V-Al_2_O_3_
Carbon-Epoxy	AlN-Mo	-	Graded SS 316L
SiCw-6061	-	-	-
Al-alloy-CNT	-	-	-

**Table 2:** The operation conditions of direct energy deposition (DED).

Parameter	Value
Average laser power (SS 316L), W	308
Average laser power (bronze), W	500
Maximal laser power (SS 316L), W	450
Maximal laser power (bronze), W	750
Scanning speed, m/min	0.85
Powder rate, g/min	3.5
Coaxial gas flow, L/min	9.0
Powder gas flow, L/min	2.0
Shield gas flow, L/min	10.0
Cooling time between layers, s	5
s, µm	800
c, µm	300

## Appendix A

In the equation, let us change the variable *dC_l_*/*dx*′ = *U*:

(A1) $$D\frac{dU}{{dx}^{\prime }}+v_{cryst}\cdot U=0$$

(A2) $$\frac{D}{v_{cryst}}\cdot \frac{dU}{dx^{\prime }}=-U$$

Separating the variables in (A2),

(A3) $$-\frac{D}{v_{cryst}}\cdot \int \frac{dU}{U}=\int dx^{\prime }$$

After integrating,

(A4) $$c_{1}+x^{\prime }=-\frac{D}{v_{cryst}}\cdot lnU$$

where $c_{1}=const$. Let us perform a back substitution:

(A5) $$c_{1}+x^{\prime }=-\frac{D}{v_{cryst}}\cdot ln\frac{dC_{l}}{dx^{\prime }}$$

Exponentiating Equation (A5),

(A6) $$\frac{dC_{l}}{dx}=e^{-\frac{v_{cryst}\cdot \left(c_{1}+x^{\prime }\right)}{D}}\;\;\mathrm{so}$$

(A7) $$C_{l}=\int e^{-\frac{v_{cryst}}{D}\cdot \left(c_{1}+x\right)}\cdot dx=-\frac{D}{v_{cryst}}\cdot e^{-\frac{v_{cryst}}{D}\cdot \left(c_{1}+x\right)}+c_{2}$$

where $c_{2}=const$. The boundary conditions are as follows:

(A8) $$\{\begin{array}{c} C_{l}=C_{0}\;if\;x^{\prime }=\infty \\ C_{l}=\frac{C_{0}}{k}\;if\;x^{\prime }=0 \end{array}$$

where *C*_0_ is an initial admixture concentration [52]. From the first condition,

(A9) $$c_{2}=C_{0}$$

From the second condition, (A9) and (A7):

(A10) $$C_{l}=\frac{C_{0}}{k}=-\frac{D}{v_{cryst}}\cdot e^{-\frac{v_{cryst}}{D}\cdot C_{1}}+C_{0}$$

(A11) $$e^{-\frac{v_{cryst}}{D}\cdot C_{1}}=\frac{v_{cryst}}{D}\cdot \left(C_{0}-\frac{C_{0}}{k}\right)=\frac{C_{0}\cdot v_{cryst}}{D}\cdot \left(\frac{k-1}{k}\right)$$

Let us apply $e^{-\frac{v_{cryst}}{D}\cdot C_{1}}$ from (A11) to (A7):

(A12) $$C_{l}=\frac{D}{v_{cryst}}\cdot e^{-\frac{v_{cryst}}{D}\cdot x^{\prime }}\cdot e^{ln\left(\frac{v_{cryst}}{D}\cdot C_{0}\cdot \frac{k-1}{k}\right)}+C_{0}$$
